# Convenient and Efficient Microwave-Assisted Synthesis of a Methyl Derivative of the Fused Indoloquinoline Alkaloid Cryptosanguinolentine

**DOI:** 10.3390/molecules15053171

**Published:** 2010-04-29

**Authors:** Robert M. Gengan, Pitchai Pandian, Chandraprakash Kumarsamy, Palathurai S. Mohan

**Affiliations:** 1 Department of Chemistry, Steve Biko Campus, Durban University of Technology, Durban-4001, South Africa; 2 Department of Chemistry, School of Chemical Sciences, Bharathiar University, Coimbatore-641046, Tamil Nadu, India; E-Mail: ps_mohan_in@yahoo.com (P.S.M.)

**Keywords:** cryptosanguinolentine, microwave, phase transfer catalysis

## Abstract

An efficient synthesis of a methyl derivative of the indoloquinoline alkaloid cryptosanguinolentine based on microwave-assisted reactions is described. The microwave-assisted synthesis of an intermediate 4-hydroxy-2-methylquinoline yielded 86% of the desired product and other intermediates prepared yielded high % of products in shorter reaction times, under optimum conditions, as compared to traditional methods.

## 1. Introduction

The roots of *Cryptolepis sanguinolenta*, a plant indigenous to West Africa [1,2] have been used by Ghanian traditional healers to treat various disorders, including outbreaks of fever due to malaria and urinary tract infections [3,4,5]. The efficacy of a concoction of the roots stimulated researchers to isolate the organic components present, characterize their structures and elucidate their biological activities. Alkaloids such as the angular fused indoloquinoline alkaloid cryptosanguinolentine and the linear indoloquinoline alkaloids cryptolentine and cryptotackieine were isolated and characterized [6]. These compounds exhibit strong antiplasmodial activity and behave as DNA intercalating agents, thereby inhibiting DNA replication and transcription. Crypto-sanguinolentine, a planar aromatic compound with functional groups at positions suitable for intercalating with a DNA helix and thereby altering the conformation of this helix [6] has received significant attention. Methyl derivatives of these types of indoloquinoline alkaloids have been reported [7,8] to act as cytotoxic agents, liposomally-formulated anticancer agents [9], and DNA-topoisomerase II inhibitors [10].

According to literature, the synthesis of cryptosanguinolentine by an intramolecular reaction of an iminophosphorane with an isocyanate [11,12,13], by the regioselective thermocyclization of the corresponding azide [14] or by *ortho-*metalation using a cross-coupling strategy [15] was used in the 1990s. Our research thrust in the synthesis of cryptosanguinolentine was initially based on a three step photo-induced cyclization reaction giving excellent yield [16]. We subsequently developed a convenient and efficient three step procedure using the Fischer indole strategy [17] which gave the products in high yield. The Fischer indole methodology is one of the most important processes in heterocyclic chemistry, leading to a large variety of hetero-polycyclic biologically active compounds containing the indole nucleus and in some cases is the only method available for reaching target structures. Our subsequent report on the synthesis of the methyl derivative of cryptosanguinolentine was *via* a heteratom-directed photoannulation technique [18]. Recently we reported a five step procedure using a simple starting material, viz., β anilinocrotonate, with the aid of a photochemical reactor and a domestic microwave [19]. In continuation with the synthesis of the methyl derivative of cryptosanguinolentine we herein report a more efficient synthetic scheme using microwave assisted reactions to produce high yields of the intended products. The main benefits of performing reactions under microwave conditions are the significant enhancement of reaction rates, higher product yields as well as conforming to global demands for the use of green technology.

## 2. Results and Discussion

**Scheme 1:**
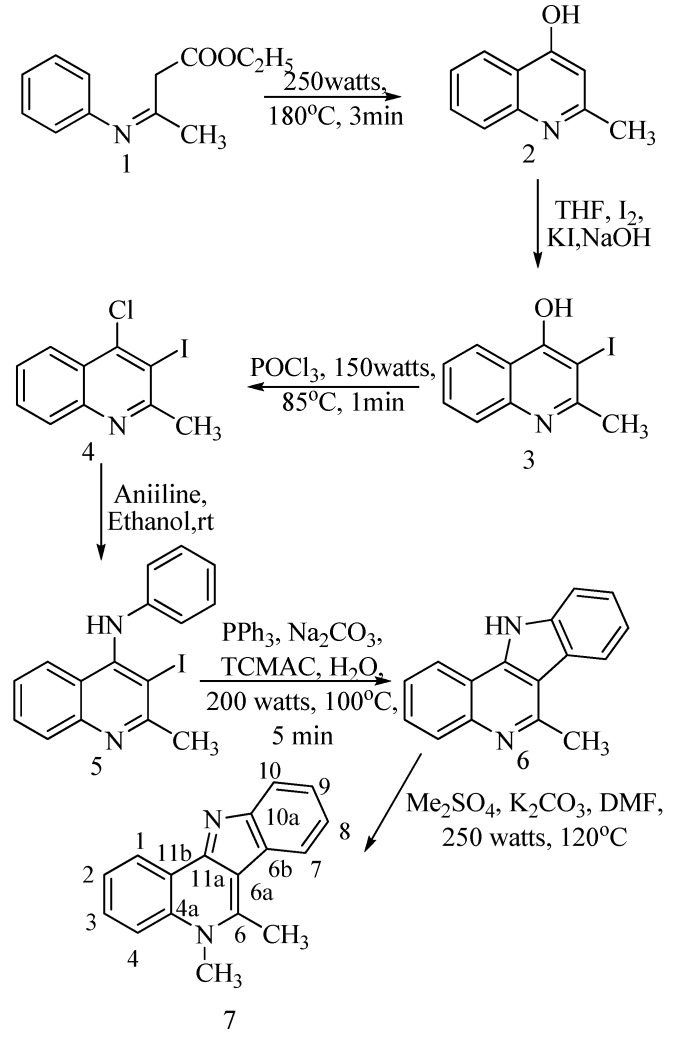
Synthesis of 5,6-dimethyl-5*H*-indolo[3,2-*c*]quinoline 7 from β-anilinocrotonate 1.

The optimum microwave conditions for the preparation of 4-hydroxy-2-methylquinoline (**2**) from β-anilinocrotonate (**1**) (Scheme 1) were optimised; compound **1 **was heated directly without any solvent for three minutes at 250 watts and 180 ºC. The solid was collected, purified and the yield recorded as 86%. Compound **2 **was confirmed by its IR spectrum, which exhibited a broad characteristic O-H stretch at 3,062 cm^-1^and CH_3 _C-H stretching at 2,767 cm^-1^; its ^1^H-NMR spectrum displayed a CH_3 _singlet at δ 2.49, and a broad singlet at δ 11.60 for the O-H proton. Our earlier investigations [19] of the yield of **2 **via a domestic microwave was marginally lower (80%).

3-Iodo-4-hydroxy-2-methylquinoline (**3**) was prepared from the reaction of **2** with a mixture of iodine, potassium iodide and sodium hydroxide in tetrahydrofuran. The product identity was confirmed from its ^1^H-NMR data by the disappearance of the singlet at δ 5.90 due to the C-3 proton of the 4-quinolone. Thereafter **3 **was refluxed with phosphorous oxychloride under microwave irradiation for 1 minute at 150 watts and 85 ºC to produce **4** in 95% yield. The yield was the same as our previous report [19], however the reaction time was significantly reduced compared to the classic solvent reflux methodology. Compound **4** was confirmed by its IR spectrum which showed the appearance of C-Cl stretching at 758 cm^-1^, as well as the disappearance of the broad OH signal. This was further confirmed by the disappearance of the OH peak in the ^1^H-NMR spectrum and the appearance of δ 110.78 for the corresponding carbon in ^13^C-NMR spectrum.

The amination of **4 **to **5 **was a simple room temperature reaction performed by stirring **4 **in a mixture of aniline in ethanol to produce a pale yellow solid **5**; this was recrystallized from chloroform and the yield recorded as 98%. The characteristic N-H stretching at 3334 cm^-1 ^in the IR spectrum, a broad singlet at δ 8.56 for the N-H proton in the ^1^H-NMR and a C_3_ signal at δ 51.97 in the ^13^C-NMR spectrum confirms the identity of compound **5**.

For the next step in the reaction scheme, compound **5** was taken in up water and mixed with triphenylphosphine, sodium carbonate, palladium acetate with tricaprylyl methyl ammonium chloride (TCMAC) and was refluxed at 80 ºC under microwave irradiation. The spectral data of **6 **matched the compound obtained from the photochemical cyclization method we reported recently [19]; the elimination of iodine due to ring closure was confirmed by the appearance of a C-3 signal at δ 126.73 in the ^13^C-NMR spectrum. The yield (83%) of **6 **washigher than that obtained by the photochemical procedure. To our knowledge, this is the first time this cyclization reaction has been performed. The improved yield is due to the eco-friendly methodology that we used: a microwave assisted reaction using water as the solvent, phase transfer catalysis, and palladium acetate catalysis for a regio- and stereoselective product.

The final step in the synthesis of the methyl substituted cryptosanguinolentine **7** involved selective methylation of the *N* atom of the quinoline skeleton. This was accomplished by reacting **6 **with dimethyl sulphate and potassium carbonate under microwave conditions (250 watts, 120 ºC for 3 min). The yield of **7** however, was marginally higher than in our previous report [19]. The methylation product was confirmed by the appearance of a singlet at δ 3.75 in the ^1^H-NMR spectrum.

## 3. Experimental Section

### 3.1. General methods

Melting points are uncorrected. Infrared spectra were recorded on a Perkin-Elmer Paragon 1000 FTIR spectrophotometer as potassium bromide discs, unless otherwise indicated. **^1^**H-NMR spectra were obtained on a Bruker 500 MHz, 400 MHz or 300 MHz instrument in either CDCl**_3_** or DMSO-D_6 _solutions using tetramethylsilane as an internal standard. *J *Values are given in Hz. Mass spectra were obtained at the Indian Institute of Chemical Technology, Hyderabad, India. Column chromatography utilized Merck silica gel 60 and hexane and ethyl acetate as eluants. All the basic chemicals were purchased from Merck (South Africa) and Fluka (South Africa). The Microwave used is a CEM Microwave synthesizer.

### 3.2. Preparation of β-anilinocrotonate (**1**)

Freshly distilled aniline (0.25 mol) and ethyl acetoacetate (0.25 mol) were mixed, 5–10 drops of concentrated hydrochloric acid was added and the mixture was gently shaken at room temperature. This mixture was left aside and within a few minutes it became turbid thereby indicating the liberation of water due to a condensation reaction. At this stage, the mixture was placed inside a vacuum desiccator over concentrated sulphuric acid and kept for 2–3 days. A deep yellow oily liquid **1**, was separated and dried over anhydrous sodium sulphate.

### 3.3. Synthesis of 4-hydroxy-2-methylquinoline (**2**)

Dried ethyl-β-anilinocrotonate (**1, **25 mL) was subjected to microwave heating for 3 min at 250 watts and 150 ºC in the synthetic microwave oven. The solid formed was washed with ethyl acetate (200 mL), followed by a mixture of chloroform and petroleum ether (30:10) to give a pure white powder. Yield 11.82 g (86%); m.p. 256 ºC; IR (KBr) [υ_max_ cm^-1^] 3062 (O-H), 2767 (C-H); ^1^H-NMR (DMSO-D_6_, 300 MHz, DMSO-D_6_ = 2.50 ppm) δ 2.49 (s, 3H, CH_3_), δ 5.90 (s, 1H, H-3), δ 7.26 (t, 1H, H-6), δ 7.49 (d,1H, H-5, *J = *7.2 Hz), δ 7.60 (t, 1H, H-7, *J = *7.6 Hz), δ 8.02 (d,1H, H-8, *J = *8.0 Hz), δ 11.60 (bs, 1H, OH).

### 3.4. Synthesis of 4-hydroxy-3-iodo-2-methylquinoline (**3**)

A mixture of 2-phenylquinolin-4(1H)-one 2 (0.5 g, 2.26 mmol), iodine (1.15 g, 4.52 mmol) and sodium carbonate (0.36 g, 3.43 mmol) in THF (20 mL) was stirred at room temperature for 12 h and then poured into a saturated ice-cold aqueous sodium thiosulphate solution. The precipitate was collected, washed with water and recrystallized to afford compound 3. Yield 0.920 g (75%); m.p. 190 ºC; IR (KBr)[υ_max_ cm^-1^] 3257 (O-H), 2916 (C-H); ^1^H-NMR (DMSO-D_6,_ 400 MHz, DMSO-D_6_ = 2.50 ppm) δ 2.49 (s, 3H, CH_3_), δ 7.37 (t, 1H, C_6_-H, *J = *7.2 Hz), δ 7.55 (d, 1H, H-5, *J = *8.4 Hz), δ 7.69 (t, 1H, H-7, *J = *8.0 Hz), δ 8.09 (d, 1H, H-8, *J* = 8.0 Hz), δ 12.20 (s, 1H, OH).

### 3.5. Synthesis of 4-chloro-3-iodo-2-methylquinoline (**4**)

A mixture of 4-hydroxy-3-iodo-2-methylquinoline (**3**, 1.43 g, 0.005 mol) in phosphorus oxychloride (4 mL) was heated under reflux for 1 min at 150 watts and 80 ºC. The mixture was cooled to room temperature, slowly added to ice-water and neutralized with a dilute NaOH solution. The precipitate thus obtained was filtered and dried. Yield 1.42 g (95%); m.p. 70 ºC; IR (KBr) [υ_max_ cm^-1^] 1550 (C=N), 1338 (C-I), 758 (C-Cl); ^1^H-NMR (CDCl_3_, 400 MHz, CDCl_3_ = 7.24 ppm) δ 3.02 (s, 3H, CH_3_), δ 7.56 (t, 1H, H-6, *J* = 7.2 Hz), δ 7.74 (t, 1H, H-7, *J = *8.0 Hz), δ 8.00 (d, 1H, H-5, *J = *8.0Hz), δ 8.20 (d, 1H, H-8, *J* = 8.0Hz); ^13^C-NMR (CDCl_3_, 100 MHz, CDCl_3_ = 70.00 ppm) δ 155.13 (C-2), 27.34 (CH_3_), 110.78 (C-3), 157.50 (C-4_)_, 131.77 (C-4a), 120.45 (C-5), 124.33 (C-6), 127.62 (C-7), 129.15 (C-8), 149.34 (C8a), 144.79 (C-1´), 119.42 (C-2´ & C-2´´), 115.71(C-3´& C-3´´), 120.11 (C4´).

### 3.6. Synthesis of 4-anilino-3-iodo-2-methylquinoline (**5**)

4-Chloro-3-iodo-2-methylquinoline (**4**, 1.51 g, 0.005 mol) and aniline (0.93 mL, 0.01 mol) were dissolved in dry ethanol (30 mL). The initial colour of the reaction mixture was colourless, but changed into yellow after some 15 min. The reaction was monitored by thin layer chromatography (tlc) and the formation of product was confirmed by the appearance of a new spot with Rf equal to the standard. The reaction mixture was stirred for a further 30 min, filtered, dried and recrystallized from chloroform. Yield 1.29 g (98%); m.p. 180 ºC; IR (KBr) [υ_max_ cm^-1^] 3334 (N-H); ^1^H-NMR (CDCl_3_, 400 MHz, CDCl_3_ = 7.24 ppm) δ 3.18 (s, 3H, CH_3_), δ 7.07 (d, 2H, Ph-H-2′& H-2′′, *J = *8.0 Hz), δ 7.22 (t, 2H, H-3′& H-3′′, *J* = 8.0 Hz), δ 7.38 (m, 3H, H-5, H-6 & H-7, *J = *8.0 Hz), δ 7.54 (d, 1H, H-8, *J = *8.0Hz), δ 7.72 (t, 1H, H-4′, *J* = 7.2Hz), δ 8.56 (bs, 1H, NH); ^13^C-NMR (CDCl_3_, 100 MHz, CDCl_3_ = 70.00 ppm) δ 159.01 (C-2), 13.05 (CH_3_), 51.97 (C-3), 148.27 (C-4), 125.16 C-4a)-, 124.33 (C-6), 129.76 (C-7 & C-8_)_, 147.39 (C-8a), 131.08 (C-1´), 96.43 (C-2´& C-2´´), 129.46 (C-3´ & C-3´´), 125.82 (C-4´& C-5).

### 3.7. Synthesis of 6-methyl-11H-indolo[3,2-c]quinoline (**6**)

A mixture of compound **5** (1.96 g, 0.0024 mol), triphenylphosphine (0.12 g, 0.0046 mol), palladium(II) acetate (0.03 g, 0.0001 mol) and sodium hydrogen carbonate (0.62 g, 0.0074 mol) and tricaprylmethylammonium chloride as phase transfer catalyst was refluxed in water at 100 ºC for 5 min at 200 watts. The mixture was allowed to cool to room temperature, poured into water and then acidified to pH 2–3 with dilute hydrochloric acid. The mixture was extracted several times with ethyl acetate and the combined organic extracts were washed with water, dried with anhydrous sodium sulphate, filtered and evaporated under reduced pressure giving 6-methyl-11*H*-indolo[3,2-*c*]quinoline (**6**). Yield 0.95 g (83%); m.p. 210 ºC; IR (KBr)[υ_max_ cm^-1^] 3307 (N-H); ^1^H-NMR (DMSO-D_6,_ 500 MHz, DMSO-D_6_ = 2.50 ppm) δ 2.43 (s, 3H, CH_3_), δ 7.33–7.88 (m, 7H, Ar-H), δ 8.07 (d, 1H, H-4), δ 10.51 (bs, 1H, NH); ^13^C-NMR (DMSO-D_6,_ 125 MHz, DMSO-D_6_ = 40.0 ppm) δ 127.41 (C-1), δ 124.69 (C-2), δ 126.73 (C-3), δ 129.44 (C-4), δ 150.17 (C-4a), δ 154.75 (C-6), δ 23.14 (CH_3_), δ 122.57 (C-6a), δ 126.78 (C-6b), δ 120.29 (C-7), δ 124.43 (C-8), δ 119.54 (C-9), δ 118.64 (C-10), δ 135.67 (C-10a), δ 136.73 (C-11a), δ 130.11 (C-11b).

### 3.8. Synthesis of 5,6-dimethyl-5H-indolo[3,2-c]quinoline(**7**)

A mixture of compound **6** with dimethyl sulphate (1.5 mL) was taken up in dimethylformamide (10 mL) and potassium carbonate (500 mg) was added. The whole mixture was subjected to microwave heating for 3 min at 250 watts and 120 ºC. The completion of the reaction was monitored by tlc. The mixture was then poured into crushed ice (300 mL) and extracted with ethyl acetate (100 mL × 3). The combined organic layers was subjected to silica gel column chromatography with a gradient elution with petroleum ether and ethyl acetate in a 35:65 ratio, and the mass of 5,6-dimethyl-5*H*-indolo[3,2-*c*]quinoline (**7**) was recorded. Yield 1.08 g (86%); m.p. 189 ºC; IR (KBr)[υ_max_ cm^-1^] 1626 (C=N) ; ^1^H-NMR (DMSO-D_6,_ 500 MHz, DMSO-D_6_ = 2.50 ppm) δ 2.43 (s, 3H, C_6_–CH_3_), δ 3.75 (s, 3H, C_5_–CH_3_), δ 7.36–7.92 (m, 7H, Ar-H), δ 8.26 (d, 1H, H-4, *J *8.2Hz); ^13^C-NMR (DMSO-D_6,_ 125 MHz, DMSO-D_6_ = 40.0 ppm) δ 127.41 (C-1), δ 124.69 (C-2), δ 126.73 (C-3), δ 129.44 (C-4), δ 150.17 (C-4a), δ 154.75 (C-6), δ 23.14 (CH_3_), δ 122.57 (C-6a), δ 126.78 (C-6b), δ 120.29 (C7), δ 124.43 (C-8), δ 119.54(C-9), δ 118.64 (C-10), δ 135.67 (C-10a), δ 136.73 (C-11a), δ 130.11 (C-11b).

## 4. Conclusions

The use of microwave irradiation provided an efficient, rapid and practical method for the synthesis of a methyl derivative of the indoloquinoline alkaloid cryptosanguinolentine. Higher yields were obtained for all intermediates as well as the final product.
